# The Toll-Like receptor adaptor TRIF contributes to otitis media pathogenesis and recovery

**DOI:** 10.1186/1471-2172-10-45

**Published:** 2009-08-05

**Authors:** Anke Leichtle, Michelle Hernandez, Kwang Pak, Nicholas J Webster, Stephen I Wasserman, Allen F Ryan

**Affiliations:** 1 Department of Surgery/Otolaryngology University of California, San Diego, 9500 Gilman Avenue, La Jolla, California 92093, USA; 2 Department of Medicine/Rheumatology, Allergy and Immunology University of California, San Diego, 9500 Gilman Avenue, La Jolla, California 92093, USA; 3 Department of Medicine/Endocrinology, University of California, San Diego, 9500 Gilman Avenue, La Jolla, California 92093, USA; 4 Department of Pediatrics, Division of Allergy, Immunology, Rheumatology, & Infectious Diseases, University of North Carolina at Chapel Hill School of Medicine, 4030 Bondurant Hall, CB#7000, Chapel Hill, NC 27599, USA; 5 Department of Otolaryngology, University of Lubeck, Ratzeburger Allee 160, Lubeck 23538, Germany

## Abstract

**Background:**

Toll-like receptor (TLR) signalling is crucial for innate immune responses to infection. The involvement of TLRs in otitis media (OM), the most prevalent childhood disease in developed countries, has been implicated by studies in middle ear cell lines, by association studies of TLR-related gene polymorphisms, and by altered OM in mice bearing mutations in TLR genes. Activated TLRs signal via two alternative intracellular signaling molecules with differing effects; MyD88 (Myeloid differentiation primary response gene 88) inducing primarily interleukin expression and TRIF (Tir-domain-containing adaptor inducing interferon β) mediating type I interferon (IFN) expression. We tested the hypothesis that TRIF and type I IFN signaling play a role in OM, using a murine model of OM induced by non-typeable *Haemophilus influenzae *(NTHi). The ME inflammatory response to NTHi was examined in wild-type (WT) and TRIF-/- mice by qPCR, gene microarray, histopathology and bacterial culture.

**Results:**

Expression of TRIF mRNA was only modesty enhanced during OM, but both type I IFN signalling genes and type I IFN-inducible genes were significantly up-regulated in WT mice. TRIF-deficient mice showed reduced but more persistent mucosal hyperplasia and less leukocyte infiltration into the ME in response to NTHi infection than did WT animals. Viable bacteria could be cultured from MEs of TRIF-/- mice for much longer in the course of disease than was the case for middle ears of WT mice.

**Conclusion:**

Our results demonstrate that activation of TRIF/type I IFN responses is important in both the pathogenesis and resolution of NTHi-induced OM.

## Background

Otitis media (OM) is the most common pediatric disease in industrialized nations [1,2]. OM is characterized by hyperplasia of the middle ear (ME) mucosa, the development of effusion, and leukocytic infiltration of the ME [3,4]. The etiology of OM is multifactorial, with Eustachian tube dysfunction, prior viral infection and allergy [5-7] all being factors known to contribute to OM incidence. However, bacterial infection is common to most acute and/or recurrent OM. Non-typeable *Haemophilus influenzae *(NTHi), which since the introduction of pneumococcal vaccines is associated with an increasing percentage of OM [8], is one of the most common pathogens isolated from the ME in OM.

**Figure 1:**
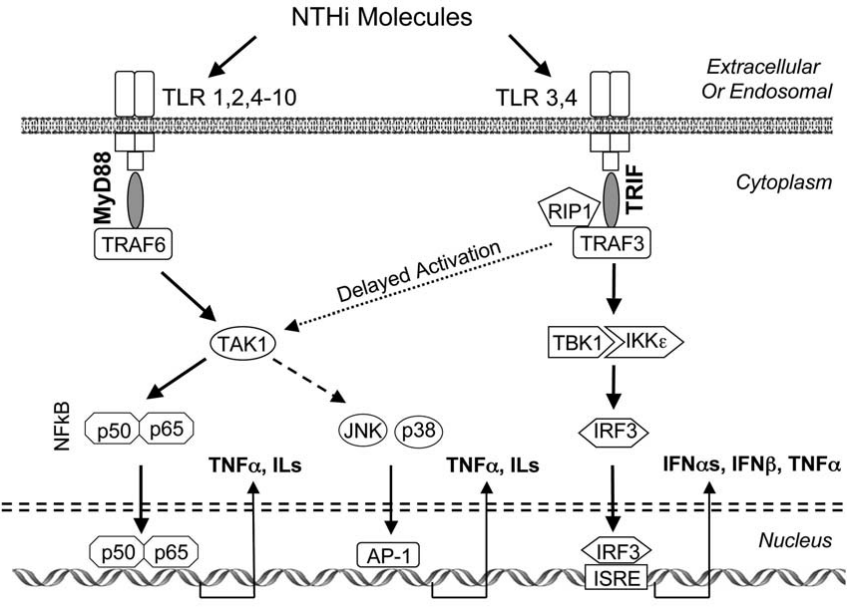
**A schematic representation of TLR signaling via the MyD88 versus TRIF adaptors**. MyD88 signaling strongly stimulates the production of pro-inflammatory interleukins, primarily via NFκB. A subset of TLRs signal via the adaptor TRIF, resulting primarily in the production of type I IFNs. The role of TRIF signaling in OM, previously unknown, is explored in this study. TLR, Toll-like receptor; MyD88, Myeloid differentiation primary response gene 88; TRIF, Tir-domain-containing adaptor inducing interferon β; TRAF, TNF-receptor-associated factor; RIP1, receptor interacting protein kinase 1; TAK1, mitogen-activated protein kinase kinase kinase 7; TBK1, TANK-binding kinase 1; IKKε, inhibitor of kappa light polypeptide gene enhancer in B-cells, kinase epsilon; p50, NFκB, nuclear factor of kappa light polypeptide gene enhancer in B-cells; p50, NFκB, subunit 1; p65, NFκB, subunit 3; JNK, Jun kinase; p38, p38 mitogen activated protein kinase IRF3, interferon regulatory factor 3; ISRE, interferon-stimulated response element; TNFα, tumor necrosis factor alpha; IL, interleukin; IFN, interferon.

NTHi can influence host cells through interaction with pattern recognition receptors, which recognize molecules produced by pathogens and which play a key role in innate immunity. They serve as the first line of host defense in infection, and they include the family of Toll-like receptors (TLRs). As illustrated in Figure 1, TLR activation induces pro-inflammatory cytokines such as interleukins (ILs), tumor necrosis factor alpha (TNFα) and type I interferons (IFNs), via signaling cascades including those dependent upon the MyD88-NFkB, TRIF-IRF3 and/or MAP kinase pathways. All TLRs except TLR3 recruit the adaptor MyD88 (Myeloid differentiation primary response gene 88), which can influence gene expression by activating downstream intracellular pathways. The most dominant of these is NFκB, leading to the production of TNFα and other pro-inflammatory cytokines. MyD88 can also activate the JNK and p38 MAP kinase pathways with subsequent expression of stress and inflammation genes. Finally, MyD88 can activate the IFN response factors (IRFs) IRF5 and IRF7. IRF5 mediates the production of pro-inflammatory cytokines, while IRF7 can lead to the expression of type I IFN genes (IFNs α and β). The alternative TLR adaptor TRIF (Tir-domain-containing adaptor inducing interferon β) is recruited only by TLR3 and TLR4. TRIF primarily activates type I IFN gene expression via IRF3, although via RIP1 and with delayed kinetics it can activate NFκB and MAPKs [9-11].

While ME responses to TLR ligands such as peptidoglycans and lipopolysaccharide (LPS) are well documented [12,13], the role played by TLR signaling pathways in OM pathogenesis has not been as fully studied. In ME epithelial cell lines, NTHi induces TLR2 expression [14,15] and TLR2 activation regulates the expression of pro-inflammatory cytokines and mucin genes [16-18]. Moreover, polymorphisms in the TLR4 gene are associated with increased OM susceptibility in children [19]. Polymorphisms in the genes for TNFα, a major pro-inflammatory target of TLR signaling, and for IL-10, which often opposes TLR signaling, are associated with OM [19,20]. Experimentally, reduced short-term responses to NTHi, from 6 hours to 3 days, were reported in the MEs of C3H/HeJ mice, which express a nonfunctional TLR4 [21]. TLR signaling deficiencies have also been shown to induce abnormalities in the recovery from bacterial infection at other sites [22,23].

We recently reported that MyD88 deficiency significantly prolongs OM and delays bacterial clearance from the ME of mice by weeks [24], suggesting that this adaptor molecule is critical for OM recovery. However, OM does improve over time in MyD88-deficient animals, with the majority of MEs clearing NTHi by 42 days (our unpublished observation), suggesting a MyD88-independent response to infection in the ME. We also evaluated OM in TLR4-deficient mice, and found impaired early bacterial clearance and delayed recovery [25]. In dendritic cells, both TRIF and MyD88 are known to play major roles in responses to TLR4 ligands [26,27]. We therefore explored the potential role of the major MyD88-independent TLR signaling pathway, which acts via the adaptor protein TRIF, in OM.

Using a well-established experimental model of OM induced by NTHi [28], we evaluated the expression of genes related to TRIF signaling during acute OM, to determine whether genes that subserve this pathway or are its targets are altered, which would suggest a role in mediating innate immunity in this condition. Using TRIF-deficient mice, we also assessed the role of TRIF in OM pathogenesis and ME bacterial clearance. Compared to control mice, we demonstrate extensive expression of genes related to TRIF-mediated signaling in OM in WT mice. We also found that the lack of TRIF reduces and delays the expression of the characteristic histopathological findings of OM, indicating a role in mediating pathogenesis. We also observed delayed bacterial clearance suggesting that TRIF contributes, at least in part, to antibacterial responses in the ME. This paper presents the first demonstration of the role of TRIF in OM.

## Methods

### Animals

TRIF-/-, TLR2-/- and TLR4-/- mice on a C57BL/6 background (the null alleles were 6 × backcrossed onto C57BL/6, and then intercrossed to establish the homozygous lines) were originally generated by Beutler and colleagues [29,30], and used with their permission, but were generously supplied by Dr. Timothy Bigby of UCSD. Age-matched C57BL/6J control mice, and C57BL/6J:CB F1 hybrids for gene array analysis, were purchased from Jackson Laboratories (Bar Harbor, ME). All experiments were performed according to NIH guidelines and approved by the Institutional Animal Care and Use Committee of the San Diego VA Medical Center. The mice were housed and maintained in accordance with NIH policy for the human treatment of animal subjects.

### Bacteria

*Haemophilus influenzae *(non-typeable, biotype II; NTHi) strain 3655 (originally isolated from the ME of an otitis media patient in St. Louis; provided by Dr. Asa Melhus, Lund University) was streaked onto chocolate agar overnight. Two colonies were picked and grown in 25 ml of brain heart infusion (BHI) media with 1 ml of Fildes Enrichment (BD Diagnostic Systems). The bacteria were spun down (9,000 rpm, 10 min) and then resuspended to a final titer of 10^5 ^– 10^6^/ml [31].

### Surgery

TRIF-/- and C57BL/6J mice were divided into groups of 6 mice for each experimental time point (3 each for histopathology and bacterial culture). For DNA microarrays, 40 C57BL/6J:CB F1 hybrid mice per time point were divided into two groups. All animals were inoculated with NTHi as previously described [28,31]. Briefly, the mice were deeply anesthetized, and both ME bullae were accessed via a ventral midline incision in the neck. A hole was made in the bullae using the tip of a 21 gauge needle. Both MEs were injected with ~5 μl of NTHi inoculum, after which the incision was closed [28,31]. Uninoculated (time 0) or sham injected (saline) animals served as controls.

### qPCR

Expression of TRIF mRNA during NTHi-induced OM was assessed in individual ME mucosae from wild-type (WT), TLR2-/- AND TLR4-/- mice (n = at least 6 per time point) by qPCR s previously described [25]. The ME mucosae were dissected, mRNA was extracted and reverse transcribed, and 1 μg/μl of cDNA was amplified using commercial TaqMan qPCR primers (Applied Biosystems, Foster City, CA) for TRIF (Mm01260003_m1) and MyD88 (Mm01351743_g1) in an ABI Prism 7000 Sequence Detection System (Applied Biosystems). Fold induction was calculated using the comparative threshold cycle (Ct) method [32]. Relative expression of each target gene was normalized to levels of GAPDH and compared to uninfected mucosa.

### DNA Microarray

For each time point and condition (NTHi or PBS injection), forty WT (C57BL/6J) ME mucosae were harvested from deeply anesthetized mice at 0, 3 and 6 hours, as well as 1, 2, 3, 5 and 7 days after NTHi inoculation. The tissue from 20 different mice was pooled, to generate two independent samples. The tissue was homogenized in TRIzol™ (Invitrogen, Carlsbad, CA) and total RNA was extracted. Total RNA quality was assessed using the RNA 6000 Labchip Kit on the Agilent 2100 Bioanalyzer for the integrity of 18S and 28S ribosomal RNA. The mRNA was reverse transcribed using a T7-oligodT primer then in vitro transcribed using T7 RNA polymerase to generate biotinylated cRNA probes that were hybridized to Affymetrix MU430 2.0 microarrays. Duplicate arrays were hybridized for each time point, using RNA from pools of different mice to obtain an independent biological replication. Raw intensity data was median normalized and statistical differences in gene transcript expression levels were evaluated using a variance-modeled posterior inference approach (VAMPIRE) [33]. This program uses a Bayesian approach to identify altered genes. Statistical analysis by VAMPIRE requires two distinct steps: (1) modeling of the error structure of sample groups and (2) significance testing with *a priori*-defined significance thresholds. VAMPIRE models the existing error structure to distinguish signal from noise and identify the coefficients of expression-dependent and expression-independent variance. These models are then used to identify microarray features that are differentially expressed between treatment groups. This method allows the use of small numbers of replicates to evaluate gene expression across a continuum of conditions, down to one array per condition if (as in the present study) many samples are pooled for each array (i.e. the array itself samples the mean value) and if multiple conditions are assessed. We used two arrays per condition, as recommended by the UCSD Microarray Core. We compared mice inoculated with NTHi or PBS for each time point against uninjected (0 hour) controls to generate sets of genes that change over time with NTHi or PBS injection. We also compared mice inoculated with NTHi versus PBS at each time point to generate sets of genes that are differentially regulated between the two conditions. Bonferroni multiple testing correction (α_Bonf _< 0.05) was applied to identify only those genes with the most robust changes. Functional gene families were assessed by gene ontology (GO) analysis, and specific genes were overlaid on pathways using Genespring GX 7.3 (Agilent Technologies, Santa Clara, CA).

The IFNα's comprise a large family of highly similar genes, clustered on the same chromosome along with some pseudogenes, that are not well represented on the Affymetrix MU430 2.0 microarray: Affymetrix identifies two probes for IFNα2, two for IFNα11, three for IFNα12 and four for IFNα13. However, for human Affymetrix gene arrays, oligonucleotides for the IFNα family are reported to be incorrectly identified [34]. We therefore evaluated the IFNα probes present on the MU430 2.0 microarray by nBLAST against the mouse genome, and found similar problems with gene identification. Several IFNα probes aligned to IFNα isoforms different from those specified by Affymetrix, while other IFNα probes cross-aligned with several different IFNα's [see Additional File 1]. We therefore considered only those probes that matched a single IFNα gene. Probes were found to align uniquely to the IFNα2, 4, 5, 9, 11 and 14 genes, as well as to an unassigned IFNα gene similar to IFNα7.

As a separate evaluation of IFN signaling, we assessed the expression of IFN-responsive genes that have been identified as preferentially activated by type I IFNs (that is, IFNα's and IFNβ as opposed to IFNγ) by Baechler et al. [35], choosing the 9 genes shown in their study to be most strongly up-regulated in leukocytes by IFNβ.

As a negative control, we assessed the expression of several genes whose EST profiles on Genbank show expression highly restricted to the testis, reasoning that these genes are highly unlikely to play a role in OM: Cyct (testis cytochrome c); Tct3 (t-complex-associated testis-expressed 3); Dnajb3 (HSP40 homolog B3); Adam4 (a disintegrin and metaloprotease 4); Tcp1b (t-complex protein B) and Nkx2-6 (NK2 transcription factor-related, locus 6). None of these genes showed significant changes during OM.

As a positive control, we assessed genes reported in the literature to be strongly up-regulated in OM. All were significantly regulated in OM in the array data, at levels roughly similar to those previously reported: Muc4 (mucin 4, 5-fold at 48 and 72 hours) [36]; Muc5ac (10-fold at 24 and 48 hours) [37,38]; Muc5b (5-fold at 24 hours) [36]; S100a9 (36-fold at 24 and 48 hours), S100a8 (20-fold at 24 and 48 hours), Fth1 (ferritin heavy polypeptide 1, 3-fold at 48 hours) [39].

### Histology

Deeply anesthetized mice were perfused intracardially with 4% paraformaldehyde. MEs were harvested at 0, 6, 12 hours and 1, 2, 3, 5, 10, 14 or 21 days post inoculation, processed and sectioned. Sections were stained with haematoxylin and eosin (H&E), and micrographs were digitally recorded. Mucosal thickness and percent area of the ME lumen occupied by inflammatory cells were measured at standardized areas of the ME, and computer-averaged as described previously [28].

### Bacterial Clearance

Bacterial presence was evaluated and analyzed from at least six WT, and TRIF-/- MEs per time point, by obtaining a sample from each for NTHi culture, as described previously [24]. Briefly, a 1 μl culture loop was used to sample the ME contents. If no fluid was present, the loop was used to scrape the ME mucosa to recover any adherent bacteria. Each loop was then used to separately streak four quadrants of a chocolate agar plate, which was incubated for 24 hours prior to evaluation of bacterial colonies. Positivity was judged based upon the presence of multiple NTHi colonies (>1) on one or more quadrants of the plate. The presence of NTHi was verified by Gram staining of colony-forming units, and by negative cultures on blood agar plates versus chocolate agar plates.

### Statistics

Except for gene array values as above, data were analyzed with ANOVA using StatView statistics software with Bonferroni correction, as described elsewhere [40]. Differences were considered significant at p ≤ 0.05.

## Results

### TRIF expression is up-regulated in the ME mucosa by NTHi

**Figure 2:**
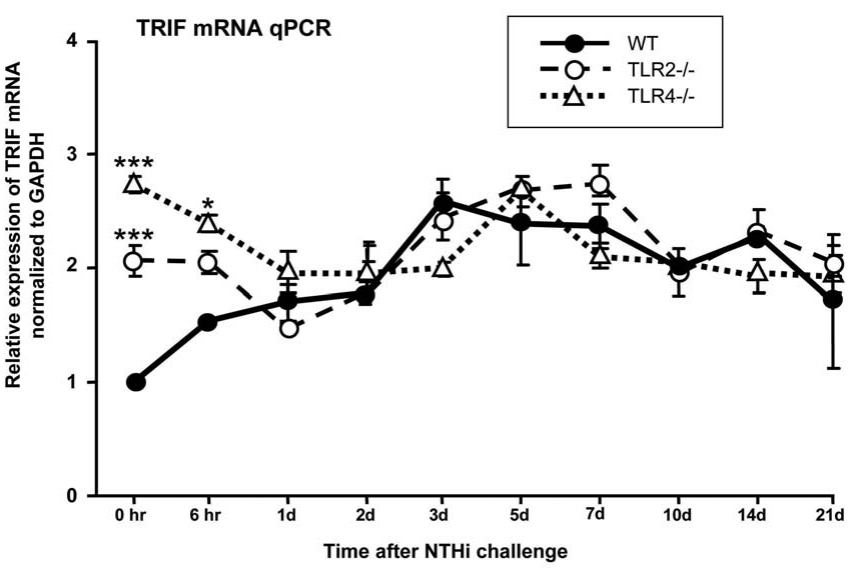
**ME expression of TRIF in WT mice, and in animals deficient in either TLR2 or TLR4, during NTHi-induced OM, assessed by qPCR**. TRIF mRNA is normalized to GAPDH mRNA, and expressed relative to that in uninfected control mice. N = 6 or more samples for each data point. Error bars represent SEM. * = P < .05; *** = P < .001.

We evaluated TRIF expression in the ME mucosa of WT mice by qPCR (Figure 2). Expression increased after administration of NTHi, reaching approximately 2.5-fold of that seen in the normal ME by 3 days after inoculation, remained elevated for 14 days, and recovered to baseline by 21 days.

### TRIF expression in the ME is altered by TLR2 and TLR4 deficiency

To evaluate TRIF expression in mice with a deficit in a major activator of MyD88, we assessed TRIF mRNA expression in TLR2-deficient mice (Figure 2A). Since TLR4 can activate both MyD88 and TRIF, we also measured TRIF mRNA in TLR4-/- mice (Figure 2A). TRIF expression in the absence of NTHi was significantly greater in both knockout strains than in uninfected WT mice. Upon infection with NTHi, TRIF mRNA decreased in these TLR deficient animals. Within 24 hours, expression in the knockout and WT mice converged, and remained similar across the three strains over the course of 21 days.

### TRIF signaling and type I IFN genes induced by NTHi infection in the ME mucosa

**Figure 3:**
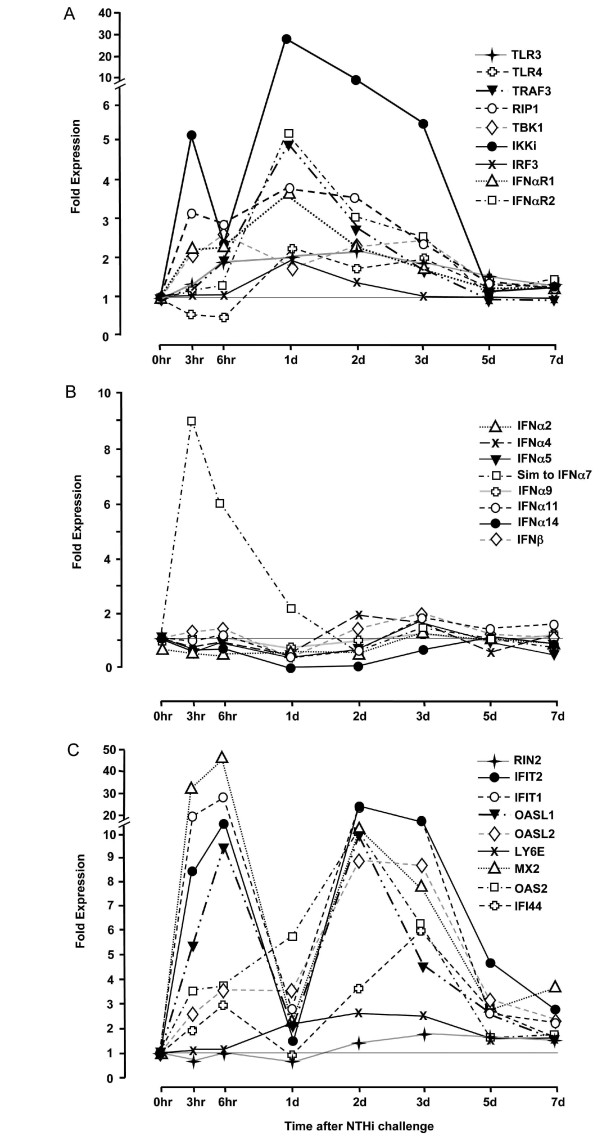
**ME expression of genes related to TRIF signaling during OM, evaluated by microarray in WT mice**. **3A. TRIF signaling genes**. mRNA encoding genes of the TRIF signaling pathway (see Figure 1) in the ME mucosa. Data are expressed as fold induction over untreated ME mucosa (0 h). Each data point represents gene arrays obtained from 2 independent sets of 20 C57BL/6J mice each. **3B. Type I IFN genes**. Not all IFNα genes are represented on the Affymetrix gene chip. However, data from those present are shown. View Within Article **3C. Type I IFN-induced genes**. A number of genes are recognized as being induced by type I IFNs. Some of these genes also have ISREs in their promoters and can be directly activated by IRFs. A set of genes previously identified as being induced by IFNβ treatment in leukocytes [35] was assessed in the ME by gene array. (Detailed data including ranges and statistical significance are provided in Additional Files 1, 2 &3.)

The expression of genes relevant to type I IFN and TRIF signaling in the ME mucosa (see Figure 1) was evaluated by gene array in C57BL/6J:CB mice (Figure 3A–C, Additional Files 2-S). GO analysis revealed that genes related to type I IFN signaling were significantly regulated during the course of OM. In addition, individual genes encoding signaling molecules associated with TRIF showed up-regulation, which was greatest at 1 day after inoculation (Figure 3A, Additional File 2). Type I IFN receptors were similarly up-regulated. In contrast, the expression of most type I IFNs represented on the array showed relatively little change, with a modest tendency for down-regulation from 3 hours to 1 day after inoculation, and slight up-regulation at 2–3 days. An exception was an unassigned IFNα gene similar to IFNα7, which was sharply up-regulated at 3 and 6 hours. (Figure 3B, see Additional File 1). Genes that are normally induced by type I IFNs were very strongly up-regulated in the ME mucosa in a biphasic manner; with peaks at 6 hours and at 2–3 days after inoculation (Figure 3C, Additional File 3). None of the genes that were differentially regulated by NTHi were significantly altered in sham (saline-injected) MEs.

### TRIF-/- mice show reduced and delayed inflammatory response to NTHi

**Figure 4:**
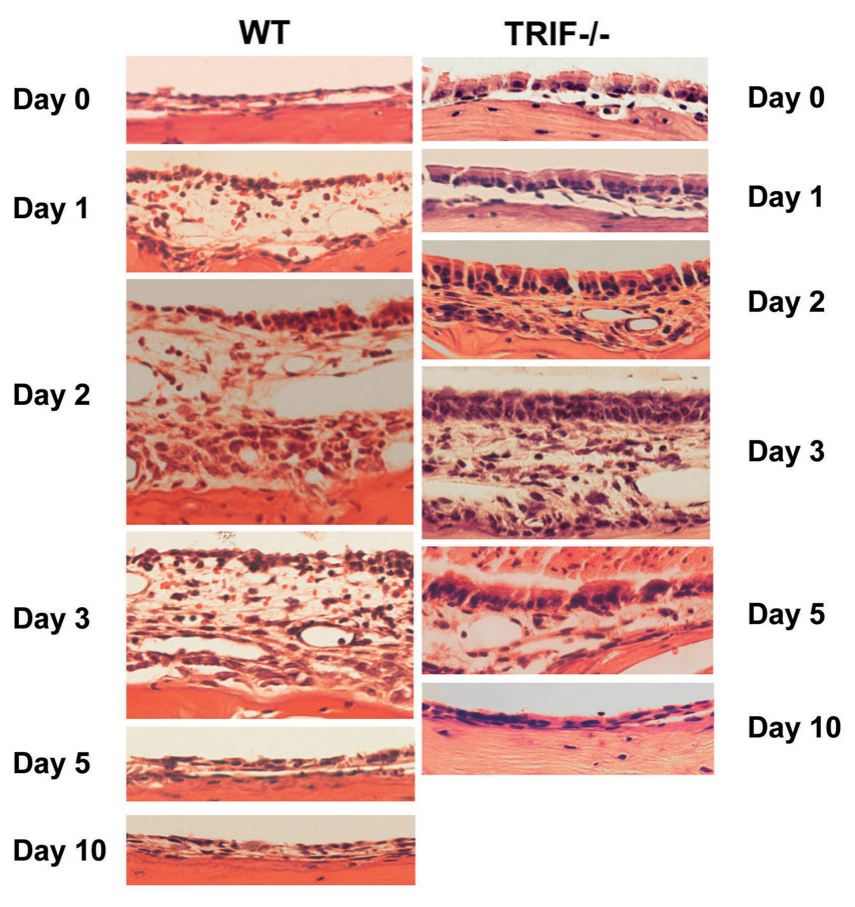
**Histological response to NTHi inoculation in the MEs of WT and TRIF-deficient mice**. Representative H&E stained sections of the ME mucosa in WT and TRIF-/- mice before (0 hr) and at various times after inoculation of the ME with NTHi.

**Figure 5:**
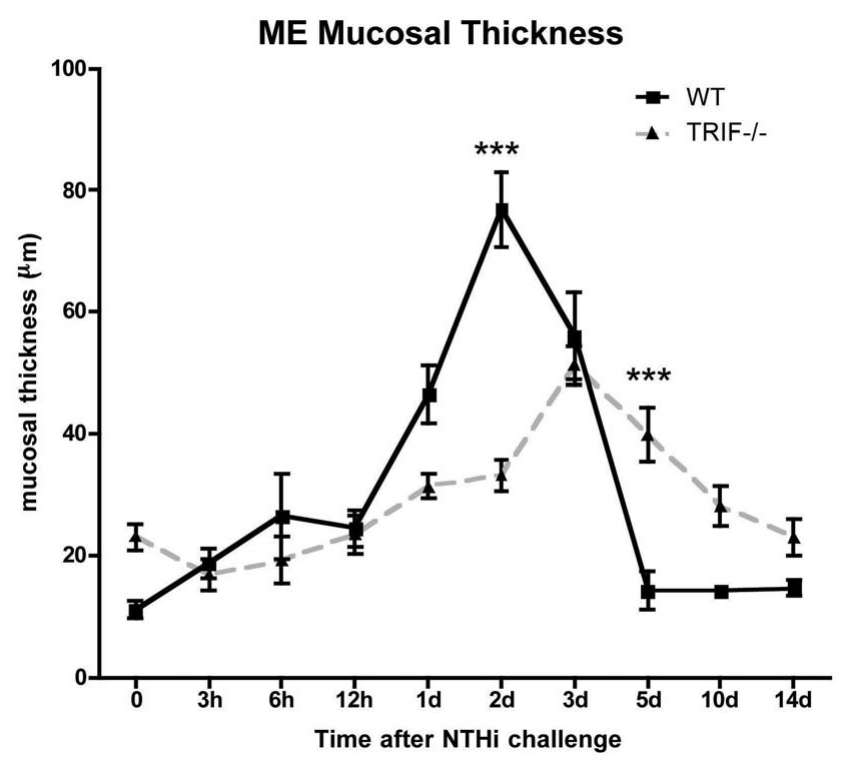
**Mucosal thickness in WT versus TRIF-/- mice during NTHi-induced OM**. Thickness of the ME mucosa, measured at standardized locations in the ME of WT and TRIF-/- mice before (0 hr) and at various times after ME inoculation with NTHi. Each data point represents the mean of 6 MEs. Bars represent one standard deviation. Significant difference between WT and TRIF-/- mice: ***P < .001.

To assess the functional influence of TRIF, we investigated the ME response to NTHi in TRIF-/- mice. WT mice demonstrated hyperplasia of the ME mucosa, which reached a maximum thickness 2 days after inoculation with NTHi (Figures 4 and 5). Mucosal thickness remained elevated at 3 days, and then recovered to baseline by 5 days in WT mice. In TRIF-/- mice, there was a slightly greater thickness of the mucosa prior to NTHi administration, compared to WT mice. However, the additional increase in mucosal thickness induced by NTHi was more gradual, peaking at 3 days after inoculation, and recovered more slowly, not reaching baseline until 10–14 days. Moreover, peak thickness was less than in WT mice.

**Figure 6:**
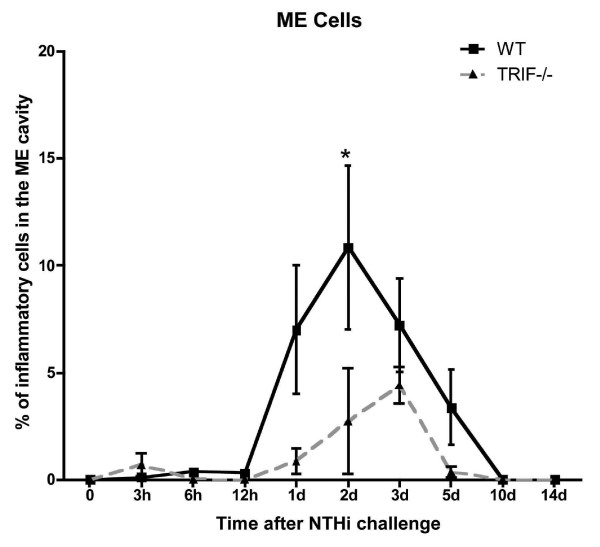
**Inflammatory cells in the ME of WT versus TRIF-/- mice during OM**. Area of the ME cavity covered by infiltrating cells, measured at standardized locations in the ME of WT and TRIF-/- mice before (0 hr) and after ME inoculation with NTHi. Each data point represents the mean of 6 MEs. Bars represent one standard deviation. Significant difference between WT and TRIF-/- mice: *P < .05.

The infiltration of leukocytes into the ME lumen of WT mice (Figure 6) was minimal until 1 day after NTHi inoculation, and like mucosal thickness, reached a maximum at 2 days. Thereafter cellular infiltration decreased to reach baseline at 10 days. The infiltration of leukocytes in TRIF-/- animals was substantially reduced and delayed compared to WT animals. However, recovery to baseline had occurred by 5 days.

### Bacterial clearance is delayed in TRIF-/- mice

Bacterial clearance of the ME cavity was examined in TRIF-deficient mice and compared to that observed in WT mice (Table 1). In WT mice, 4 out of 6 (4/6) culture plates were positive at 1 day, increasing to 6/6 at 2 days, and decreasing to 3/6 at 3 days. Thereafter, no bacteria were recovered from WT MEs. TRIF-/- MEs were similar to WT MEs for 1, 2, and 3 days after NTHi administration, with 4/6, 5/6 and 3/6 MEs positive for bacterial colonization respectively. However, in contrast to WTs, bacterial colonies could be still detected at 5 and 7 days, with 1 positive plate out of 6. Cultures were negative thereafter. By comparison, the MEs of MyD88 mice were strongly positive through 21 days (data from Hernandez et al.) [24], and even at 42 days (our unpublished observation) 1/6 MEs remained positive. TLR4-/- MEs were culture-positive until 14 days.

**Table 1:** Comparison of bacterial clearance in WT, TRIF-/-, TLR4-/- and MyD88-/- MEs.

**Time after NTHi instillation**	**C57BL/6J**	**TRIF-/-**	TLR4-/-*	MyD88-/-**
**Day 1**	4/6	4/6	4/6	6/6

**Day 2**	6/6	5/6	4/6	6/6

**Day 3**	3/6	3/6	3/6	6/6

**Day 5**	0/6	1/6	4/6	6/6

**Day 10**	0/6	1/6	3/6	6/6

**Day 14**	0/6	0/6	1/6	6/6

**Day 21**	0/6	0/6	0/6	2/6

Bacterial colonization of the culture plates from the MEs of each genotype. The number of positive plates out of 6 is provided. *TLR4-/- data from Leichtle et al., [25], and **MyD88 data from Hernandez et al. [24].

## Discussion

In this investigation we found that biological processes mediated by TRIF activation contribute to the pathogenesis of NTHi-induced OM in mice. After NTHi infection of WT mice the expression of TRIF, as well as of many TRIF-associated signaling genes and type I IFN-responsive genes, was up-regulated. Moreover, animals deficient in TRIF exhibited altered OM, consisting of delayed and reduced morphological signs of mucosal hyperplasia and inflammation as well as a delay in bacterial clearance.

As noted above, TRIF is recruited and activated following ligand binding to TLR3 and/or TLR4, both of which are up-regulated in the ME by exposure to NTHi [25]. TLR4 is expressed by epithelial cells in the ME mucosa, and also by infiltrating leukocytes [25]. While TLR3 has not been localized in the ME, it is an intracellular receptor found on endosomal membranes of phagocytic and other cells [11]. Moreover, NTHi infection produces molecules that serve as ligands for these TLRs. Lipooligosaccharide (LOS), with molecular structure closely related to LPS, can activate cellular signals via TLR4 [11,41]. TLR3 is preferentially activated by double-stranded RNA from viruses [42], and this mode of stimulation would presumably not be involved in the response to NTHi. However, bacterial RNA is also able to activate TLR3 [43,44]. In addition, host molecules released during host cell injury or death can activate TLRs. TLR3 can respond to host mRNA and DNA [45] while TLR4 can be activated by heat-shock proteins [46], both of which are released during tissue injury as is known to occur in OM [e.g. [47]]. Injection of TLR ligands, including LPS, into the ME mimics many of the pathologic changes observed in OM, including mucosal inflammation and edema, ME pressure abnormalities, and infiltrate of leukocytes into the subepithelial space and the ME lumen [12]. Thus substrates for TRIF activation are present in the ME during OM, and TLR activation independent of infection can contribute to its pathogenesis.

The dominant biological response to TRIF activation is type I IFN gene expression. The type I IFNs can be produced by a variety of cell types, unlike the so-called immune IFNs (type II, IFNγ) that are produced primarily by T-cells [48]. Type I IFN expression is induced by the activation of IKKε, TBK1 and IRF3, all of which we found to be significantly up-regulated in the ME following NTHi inoculation. IRF3 activation in turn induces the expression of type I IFNs and other genes containing ISREs (IFN-stimulated response elements) in their promoters, including a number of type I IFN-inducible genes [49]. The type I IFNs are classically associated with response to viral infection. However, it has long been noted that exposure to bacteria can increase the expression of type I IFNs and IFN-related genes [e.g. [50,51]], and that expression of IKKε, downstream from TRIF and upstream from IRF3, is enhanced by LPS [e.g. [52]]. Bacterial induction of type I IFNs is presumably related to their initial role in innate immunity and their later roles in dendritic cell, macrophage and T-cell activation. Type I IFN-inducible genes have diverse functions related to inflammation and immunity, including cellular RNA and protein metabolism, growth and differentiation, apoptosis and signal transduction [53].

The production of type I IFNs by peripheral blood leukocytes is reduced in individuals susceptible to upper respiratory infections [54,55], including OM [56]. Moreover, type I IFNs are pro-inflammatory and could contribute to OM pathogenesis. In addition, type I IFN gene expression is known to make significant contributions to adaptive immunity, which could influence late responses to NTHi. For example, type I IFNs contribute to the maturation of dendritic cells [57,58], the cross-priming of CD8 T-cells [59], and the production of IL-12 [60]. In the presence of TNFα, type I IFNs can also facilitate the development of the pro-inflammatory Th1 T-cell phenotype [61]. As a result, T-cells of both IFN-β deficient [62] and IFN-α/β receptor-deficient mice [63] show a less inflammatory, Th2 bias. As noted above, TRIF can also be associated with the production of TNFα and interleukins via NFκB activation, albeit at a lower level and with delayed kinetics when compared to MyD88 mediated processes.

Our gene expression data support the idea that TRIF signaling is active during NTHi-induced OM. TRIF mRNA was found by qPCR to increase during NTHi-induced OM. Moreover, TRIF expression was up-regulated in uninfected mice deficient in either TLR2 or TLR4, possibly suggesting a compensatory change in TLR signaling. Given that TLR2 and TLR4 can employ different signaling mechanisms (Figure 1), it is surprising that TRIF expression is so similar in the absence of either gene. However, we have previously shown [25] that TLR4 is required for the early up-regulation of TLR2 induced by NTHi, which may account for the similarity.

GO analysis of gene array expression patterns during OM identified type I IFN signaling as a pathway that is significantly regulated in the ME by exposure to NTHi. Transcripts encoding most elements of the TRIF signaling cascade are also up-regulated, especially 24 hours after NTHi inoculation. While most type I IFN genes were not extensively regulated during OM, expression of at least some appears to be enhanced. This was especially true of a proposed IFNα gene similar to IFNα7, which showed a sharp spike in fold expression within hours of NTHi inoculation, but several IFN genes were mildly up-regulated later in OM. Finally, genes responsive to type I IFNs are strongly up-regulated at 3–6 hours and again at 2–3 days after NTHi inoculation.

The relatively low degree of regulation of type I IFN genes that we observed when upstream TRIF signaling and downstream IFN-regulated genes were more extensively up-regulated is puzzling. One possible explanation is the potential for regulation of IFNα genes that are not represented on the Affymetrix mouse microarray. Alternatively, increased TRIF signaling could bypass type IFNs and interact directly with ISREs in the promoters of IFN-responsive genes. Thus it can be speculated that the increased production of "IFNα similar to IFNα7," which we observed immediately after NTHi inoculation, or of an undocumented IFNα might mediate the initial response of type I IFN-inducible genes observed at 3–6 hours (Figure 3C, Additional File 3). However, many of these genes contain ISREs in their regulatory DNA [49], and so might be directly regulated by IRF3. The later peak in expression of type I IFN-inducible genes, at 2–3 days after inoculation, could again be mediated by the type I IFNs themselves, by enhanced signaling through mildly up-regulated IFNα/β receptors and/or by direct regulation of ISREs [49] through IRF3 and other elements of the TRIF signaling cascade, which were up-regulated at 1 day. Of course, an alternate potential source of type I IFN-related gene expression after NTHi is activation of IRF7 via MyD88, which can also induce the expression of type I IFNs and IFN-inducible genes. This may be especially true for their expression later in OM. While lack of MyD88 has relatively little influence upon early OM, it has a very strong influence upon late events [24]. However, the most compelling evidence for the involvement of TRIF signaling in OM is the altered OM phenotype observed in TRIF-/- mice, consisting of decreased initial inflammatory response to NTHi, and delays in both mucosal recovery and bacterial clearance. These data suggest that TRIF contributes to both early and late events in OM.

TLR4 is activated by endotoxins, including the LOS of NTHi [12], and therefore seems the most likely TLR to activate TRIF. We have shown previously [25] that mice deficient in TLR4 show initial mucosal hyperplasia similar to that of WTs, and demonstrate early leukocyte infiltration which exceeds that seen in WTs but differ in expressing early defects in TLR2 and TNFα gene expression and in persistent hyperplasia and impaired bacterial clearance. Since TLR4 can signal via the alternative adaptor MyD88, differences between the TLR4-/- and TRIF-/- phenotypes suggest that this TLR signals via both adaptors. Finally, it has been suggested that additional pathogen receptors sensitive to bacterial molecules and using the TRIF adaptor may exist [64].

In a previous study [24], we found that in response to NTHi, animals deficient in MyD88 also initially develop mucosal hyperplasia and leukocyte infiltration of the ME similar to WT mice. Since both ME mucosal hyperplasia and leukocyte infiltration are delayed in TRIF-deficient animals, it thus seems possible that initial ME mucosal hyperplasia and leukocyte infiltration in response to NTHi are mediated in part by signaling via TRIF. Interestingly, despite the delayed and reduced leukocyte infiltration in TRIF-/- mice, these animals did clear NTHi from the ME, albeit with a delay. This suggests that the TRIF-deficient macrophages that enter the ME may well be capable of efficient phagocytosis and bacterial killing. It should be noted that mucosal hyperplasia persisted longer in TRIF-/- mice than in WTs, indicating a potential involvement of this adaptor protein in OM recovery.

Importantly, MyD88-null animals show failure of OM to resolve at 21 days post-inoculation and, indeed, higher levels of peak OM at after WT animals have completely recovered [24]. They also showed failure to clear NTHi from the ME out to 42 days (our unpublished observation). These data suggest that MyD88 may be more involved in the recovery of OM than in the initiation of pathogenesis. In another study, we noted that TLR4, which can signal via TRIF, was more involved in early OM while TLR2, which does not use TRIF, was more critical for recovery [25]. Combined with the data from the present study, our results suggest that TRIF signaling may be more involved in immediate responses during NTHi OM than is MyD88. Other investigators have noted that TLR4 responses to pathogens can occur earlier than those of TLR2 [65]. One reason for this may be the ability of TLR4 to signal via TRIF, and to induce the expression of type I IFNs [30], as well as via MyD88. The type I IFN pathway has been suggested to mediate the most immediate TLR responses to infection, followed by MyD88 signaling inducing expression of pro-inflammatory cytokines like TNFα [41].

The higher levels of TRIF mRNA expressed in TLR2-/- and TLR4-/- mice in the absence of NTHi exposure suggests that activity mediated through these receptors normally suppresses the expression of TRIF. This may be a specific and compensatory response to decreased signaling via MyD88. Alternatively, increased pathogen loads in the ME may be responsible for the increase in TRIF mRNA. The enhancement of TRIF mRNA observed in our qPCR data following NTHi inoculation is consistent with the latter possibility.

## Conclusion

The present study demonstrates that TRIF signaling is important for an appropriate host response to NTHi in the ME, and to the relevance of TLRs in the response of humans to infection [19,20,66]. These findings underscore the complex interactions of TLRs acting via both MyD88-dependent, and MyD88-independent signaling pathways in the induction and resolution of NTHi- induced OM. They further suggest the potential importance of these innate immune pathway molecules and their pathways as targets for new treatments of this important human disease.

## Abbreviations

Ct: comparative threshold; GO: gene ontology; IFN: interferon; IL: interleukin; IRF: interferon response factor; ISRE: interferon-stimulated response element; LOS: lipooligosaccharide; LPS: lipopolysaccharide; ME: middle ear; MyD88: Myeloid differentiation primary response gene 88; NTHi: non-typeable *Haemophilus influenzae*; OM: otitis media; TLR: Toll-like receptor; TNFα: tumor necrosis factor alpha; TRIF: Tir-domain-containing adaptor inducing interferon β; WT: wild-type.

## Authors' contributions

AL performed animal surgery, qPCR, bacteriology and histology procedures, and contributed to writing the manuscript. MH performed animal surgery and histology procedures. KP performed bacteriology, animal surgery and DNA microarray procedures. NJW performed DNA microarray analysis. SIW and AFR supervised the project and contributed to microarray analysis and writing of the manuscript. All authors have read and approved the manuscript, and none have any conflict of interest related to this paper.

## Supplementary Material

Additional file 1 **Type I Interferon Genes Microarray Data**. The data represent the medians, ranges and significance levels of the microarray values presented in Figure 3B. Click here for file

Additional file 2 **TRIF Signaling Genes Microarray Data**. The data represent the medians, ranges and significance levels of the microarray values presented in Figure 3A. Click here for file

Additional file 3 **Type I Interferon-Induced Genes (IFNα/β >IFNγ, ref. 35) Microarray Data**. The data represent the medians, ranges and significance levels of the microarray values presented in Figure 3C. Click here for file
