# Supplementary material for: The Toll-Like receptor adaptor TRIF contributes to otitis media pathogenesis and recovery
Source: BMC Immunol. 2009 Aug 5;10:45. doi: 10.1186/1471-2172-10-45 (PMC2736931; doi:10.1186/1471-2172-10-45)
Supplement: Additional file 1 — Type I Interferon Genes Microarray Data. The data represent the medians, ranges and significance levels of the microarray values presented in Figure 3B. [file 1471-2172-10-45-S1.doc]

**Type I Interferon Genes Microarray Data**

| Time: | 0h | 3h | 6h | 24h | 2d | 3d | 5d | 7d |
| --- | --- | --- | --- | --- | --- | --- | --- | --- |
| **IFNα2** (1423028_at) | | | | | | | | |
| Fold Exp | 0.7 | 0.5 | 0.4 | 0.5 | 0.6 | 0.3 | 0.2 | 10.2 |
| Range | 0.3-1.7 | 0.2-1.5 | 0.1-1.3 | 0.2-1.1 | 0.5-0.7 | 0.2-0.5 | 0.2-0.3 | 0.1-0.8 |
| P-Value | 0.75 | 0.66 | 0.58 | 0.54 | 0.11 | 0.26 | 0.07 | 0.45 |
| **IFNα4** (1422408_at) | | | | | | | | |
| Fold Exp | 1.0 | 0.7 | 0.9 | 0.5 | 1.9 | 1.5 | 0.7 | 1.2 |
| Range | 0.7-1.3 | 0.6-0.8 | 0.8-1.1 | 0.3-0.8 | 0.8-4.5 | 1.3-1.6 | 0.6-0.8 | 0.8-1.7 |
| P-Value | 0.91 | 0.19 | 0.77 | 0.37 | 0.60 | 0.18 | 0.26 | 0.75 |
| IFNα5 (1450614_x_at) | | | | | | | | |
| Fold Exp | 0.7 | 0.4 | 0.8 | 0.4 | 0.6 | 1.6 | 0.9 | 0.3 |
| Range | 0.4-1.8 | 0.3-0.5 | 0.3-1.9 | 0.4-0.4 | 0.2-1.8 | 0.5-4.8 | 0.3-2.3 | 0.2-0.3 |
| P-Value | 0.74 | 0.19 | 0.83 | **0.05** | 0.71 | 0.75 | 0.90 | **0.03** |
| Unassigned IFNα gene similar to IFNα7 | | | | | | | | |
| Fold Exp | 1.0 | 9.0 | 6.0 | 1.9 | 0.6 | 1.3 | 0.9 | 0.7 |
| Range | 0.7-1.3 | 5.6-14.3 | 4.6-7.7 | 1.6-2.2 | 0.4-1.0 | 1.2-1.4 | 0.9-1.0 | 0.5-1.1 |
| P-Value | 0.92 | 0.13 | 0.09 | 0.15 | 0.71 | 0.26 | 0.38 | 0.54 |
| **IFNα9** (1422406_at) | | | | | | | | |
| Fold Exp | 1.0 | 1.0 | 1.0 | 0.7 | 0.9 | 1.2 | 0.8 | 1.1 |
| Range | 0.9-1.1 | 1.0-1.1 | 0.9-1.1 | 0.6-0.7 | 0.8-1.0 | 1.1-1.3 | 0.8-0.9 | 1.0-1.3 |
| P-Value | 0.97 | 0.52 | 0.85 | 0.16 | 0.54 | 0.29 | **0.03** | 0.58 |
| IFNα11 (1422332_at) | | | | | | | | |
| Fold Exp | 1.0 | 0.9 | 1.1 | 0.4 | 0.6 | 1.6 | 1.3 | 1.4 |
| Range | 0.7 -1.3 | 0.7 -1.1 | 1.0 -1.2 | 0.3-0.5 | 0.5- 0.7 | 1.2 -2.0 | 1.3 -1.3 | 1.2-1.6 |
| P-Value | 0.90 | 0.66 | 0.56 | 0.15 | 0.20 | 0.31 | **0.00** | 0.26 |
| IFNα14 (1450593_at) | | | | | | | | |
| Fold Exp | 1.0 | 0.7 | 0.7 | 0.0 | 0.1 | 0.3 | 1.0 | 0.8 |
| Range | 0.7-1.3 | 0.7-0.8 | 0.7-0.7 | 0.0-0.0 | 0.0-0.3 | 0.3-0.3 | 1.0-1.0 | 0.8-0.8 |
| P-Value | 0.90 | 0.10 | 0.08 | **0.02** | 0.30 | 0.06 | 0.13 | 0.16 |
| **IFNαB, IFNα1, IFNα6, Unassigned IFNα gene** (1450613_x_at) | | | | | | | | |
| Fold Exp | 1.0 | 0.9 | 0.9 | 0.4 | 0.1 | 0.5 | 0.8 | 1.1 |
| Range | 0.9-1.1 | 0.8-0.9 | 0.6-1.2 | 0.2-0.7 | 0.1-0.2 | 0.4-0.6 | 0.6-1.0 | 1.0-1.4 |
| P-Value | 0.98 | 0.32 | 0.76 | 0.35 | **0.04** | 0.14 | 0.54 | 0.55 |
| **IFNαB, IFNα1, IFNα6, Unassigned IFNα gene** (1450564_x_at) | | | | | | | | |
| Fold Exp | 1.0 | 0.8 | 0.9 | 0.3 | 0.4 | 1.0 | 0.8 | 0.9 |
| Range | 0.8-1.2 | 0.8-0.9 | 0.7-1.0 | 0.2-0.5 | 0.3-0.6 | 0.9-1.1 | 0.6-0.9 | 0.7-1.1 |
| P-Value | 0.93 | 0.23 | 0.62 | 0.25 | 0.25 | 0.99 | 0.41 | 0.65 |
| IFNα1, IFNα5, IFNα6, IFNα7, IFNαB, Unassigned IFNα gene (1422404_x_at) | | | | | | | | |
| Fold Exp | 1.0 | 2.3 | 2.7 | 1.0 | 0.3 | 0.7 | 0.8 | 0.9 |
| Range | 0.9-1.1 | 2.0-2.7 | 2.4-3.0 | 0.9-1.1 | 0.1-1.0 | 0.6-0.8 | 0.7-0.8 | 0.8-0.9 |
| P-Value | 0.97 | 0.12 | 0.07 | 0.81 | 0.51 | 0.24 | 0.26 | 0.21 |
| IFNα1, IFNα6, IFNαB, Unassigned IFNα gene (1450613_x_at) | | | | | | | | |
| Fold Exp | 1.0 | 0.9 | 0.9 | 0.4 | 0.1 | 0.5 | 0.8 | 01.2 |
| Range | 0.8-1.2 | 0.8-0.9 | 0.6-1.2 | 0.2-0.7 | 0.1-0.2 | 0.4-0.6 | 0.6-1.0 | 1.0-1.4 |
| P-Value | 0.93 | 0.32 | 0.76 | 0.35 | **0.04** | 0.14 | 0.54 | 0.55 |
| IFNβ (1422305_at) | | | | | | | | |
| Fold Exp | 1.0 | 1.2 | 1.3 | 0.4 | 1.3 | 1.7 | 1.1 | 1.0 |
| Range | 0.8-1.2 | 1.0-1.4 | 1.0-1.6 | 0.3-0.5 | 1.1-1.6 | 1.5-2.1 | 1.1-1.1 | 0.9-1.2 |
| P-Value | 0.95 | 0.42 | 0.47 | 0.13 | 0.38 | 0.20 | **0.01** | 0.92 |

Type I IFN genes showed inconsistently increased expression from 1-7 days after bacterial inoculation. An unassigned IFN gene similar to IFN7 showed a brisk (but not statistically significant) increase at 3 to 6 hours after inoculation.
