# Supplementary material for: The Toll-Like receptor adaptor TRIF contributes to otitis media pathogenesis and recovery
Source: BMC Immunol. 2009 Aug 5;10:45. doi: 10.1186/1471-2172-10-45 (PMC2736931; doi:10.1186/1471-2172-10-45)
Supplement: Additional file 2 — TRIF Signaling Genes Microarray Data. The data represent the medians, ranges and significance levels of the microarray values presented in Figure 3A. [file 1471-2172-10-45-S2.doc]

# TRIF Signaling Genes Microarray Data

| Time: | 0h | 3h | 6h | 24h | 2d | 3d | 5d | 7d |
| --- | --- | --- | --- | --- | --- | --- | --- | --- |
| TLR3 | | | | | | | | |
| Fold Exp | 1.0 | 1.3 | 1.8 | 1.8 | 2.1 | 1.9 | 1.6 | 1.3 |
| Range | 1.0-1.0 | 0.9-1.9 | 1.5-2.1 | 1.8-1.9 | 1.6-2.7 | 1.8-2.1 | 1.5-1.7 | 1.2-1.4 |
| P-Value | 0.98 | 0.62 | 0.15 | **0.01** | 0.21 | 0.06 | 0.13 | 0.26 |
| TLR4 | | | | | | | | |
| Fold Exp | 1.0 | 0.6 | 0.5 | 2.1 | 1.5 | 1.8 | 1.6 | 1.5 |
| Range | 0.9-1.1 | 0.6-0.6 | 0.5-0.6 | 2.0-2.2 | 1.2-1.8 | 1.5-2.1 | 1.6-1.7 | 1.5-1.6 |
| P-Value | 0.96 | **0.01** | 0.13 | **0.05** | 0.31 | 0.18 | **0.04** | **0.03** |
| **TRAF3** | | | | | | | | |
| Fold Exp | 1.0 | 1.1 | 1.8 | 4.8 | 2.6 | 1.6 | 0.8 | 0.8 |
| Range | 0.9-1.1 | 0.7-1.8 | 1.3-2.3 | 4.1-5.6 | 1.3-5.1 | 1.0-2.5 | 0.3-2.1 | 0.3-2.0 |
| P-Value | 0.96 | 0.89 | 0.30 | 0.06 | 0.39 | 0.48 | 0.86 | 0.83 |
| RIP1 | | | | | | | | |
| Fold Exp | 1.0 | 3.1 | 2.8 | 3.8 | 3.6 | 2.5 | 1.4 | 1.4 |
| Range | 0.9-1.1 | 2.7-3.5 | 2.2-3.6 | 3.6-4.1 | 2.8-4.6 | 2.4-2.5 | 1.4-1.5 | 1.3-1.5 |
| P-Value | 0.97 | 0.07 | 0.15 | **0.03** | 0.12 | **0.01** | 0.10 | 0.15 |
| TBK1 | | | | | | | | |
| Fold Exp | 1.0 | 2.0 | 2.5 | 1.6 | 2.2 | 2.1 | 1.4 | 1.2 |
| Range | 0.9-1.1 | 1.8-2.3 | 1.9-3.3 | 1.5-1.6 | 2.1-2.3 | 1.7-2.7 | 1.4-1.4 | 1.1-1.3 |
| P-Value | 0.96 | 0.13 | 0.17 | **0.05** | **0.05** | 0.20 | **0.01** | 0.21 |
| IKKi | | | | | | | | |
| Fold Exp | 1.0 | 5.1 | 2.2 | 37.4 | 8.5 | 5.8 | 1.2 | 1.3 |
| Range | 1.0-1.0 | 3.4-7.7 | 1.7-3.0 | 36.2-38.7 | 6.9-10.4 | 4.2-8.0 | 1.0-1.5 | 0.9-2.0 |
| P-Value | 0.99 | 0.16 | 0.21 | **0.01** | 0.06 | 0.12 | **0.05** | 0.591 |
| IRF3 | | | | | | | | |
| Fold Exp | 1.0 | 1.1 | 1.1 | 1.8 | 1.4 | 1.0 | 0.8 | 0.9 |
| Range | 0.9-1.1 | 0.9-1.3 | 0.9-1.3 | 1.8-1.8 | 1.3-1.6 | 0.9-1.0 | 0.8-0.9 | 0.8-0.9 |
| P-Value | 0.98 | 0.63 | 0.66 | **0.02** | 0.19 | 0.73 | 0.20 | 0.27 |
| **IRF7** | | | | | | | | |
| Fold Exp | 1.0 | 5.1 | 5.9 | 4.3 | 12.1 | 8.2 | 2.9 | 2.2 |
| Range | 0.9-1.1 | 3.1-8.3 | 4.7-7.4 | 4.3-4.3 | 11.8-12.3 | 5.61-11.9 | 2.9-3.0 | 1.9-2.5 |
| P-Value | 0.97 | 0.19 | 0.08 | **0.00** | **0.00** | 0.11 | **0.01** | 0.11 |
| **IFNαR1** | | | | | | | | |
| Fold Exp | 0.9 | 2.1 | 2.2 | 3.6 | 2.1 | 1.7 | 1.3 | 1.3 |
| Range | 0.5-1.4 | 2.0-2.3 | 2.1-2.3 | 3.5-3.6 | 1.7-2.7 | 1.4-2.1 | 1.3-1.3 | 1.1-1.6 |
| P-Value | 0.85 | 0.07 | **0.04** | **0.01** | 0.19 | 0.20 | **0.01** | 0.36 |
| IFNαR2 | | | | | | | | |
| Fold Exp | 1.0 | 1.3 | 1.4 | 5.0 | 2.8 | 2.5 | 1.2 | 1.5 |
| Range | 0.9-1.1 | 1.1-1.5 | 1.2-1.6 | 4.7-5.3 | 2.5-3.1 | 2.5-2.6 | 1.1-1.2 | 1.3-1.8 |
| P-Value | 0.98 | 0.35 | 0.25 | **0.02** | 0.07 | **0.02** | 0.17 | 0.22 |

Expression of TRIF-related signaling genes was evaluated during acute, NTHi-induced OM in mice using Affymetrix MU430 2.0 microarrays. Significant up-regulation (**bold**) was noted especially at 24 hours after inoculation.
