# Supplementary material for: The Toll-Like receptor adaptor TRIF contributes to otitis media pathogenesis and recovery
Source: BMC Immunol. 2009 Aug 5;10:45. doi: 10.1186/1471-2172-10-45 (PMC2736931; doi:10.1186/1471-2172-10-45)
Supplement: Additional file 3 — Type I Interferon-Induced Genes (IFNα/β >IFNγ, ref. 35) Microarray Data. The data represent the medians, ranges and significance levels of the microarray values presented in Figure 3C. [file 1471-2172-10-45-S3.doc]

**Type I Interferon-Induced Genes (IFNα/β>IFNγ, ref. 35) Microarray Data**

| Time: | 0h | 3h | 6h | 24h | 2d | 3d | 5d | 7d |
| --- | --- | --- | --- | --- | --- | --- | --- | --- |
| RIN2 | | | | | | | | |
| Fold Exp | 1.0 | 0.7 | 0.9 | 0.7 | 1.3 | 1.7 | 1.7 | 1.7 |
| Range | 0.8-1.2 | 0.7-0.7 | 0.7-1.0 | 0.7-0.7 | 1.2-1.4 | 1.6-1.9 | 1.6-1.7 | 1.6-1.7 |
| P Value | 0.94 | **0.03** | 0.60 | 0.08 | 0.14 | 0.10 | **0.01** | **0.05** |
| IFIT2 | | | | | | | | |
| Fold Exp | 1.0 | 8.4 | 17.2 | 1.5 | 23.1 | 13.9 | 4.7 | 2.5 |
| Range | 0.9-1.1 | 4.2-16.8 | 12.6-23.3 | 1.2-1.9 | 16.9-31.7 | 13.4-14.4 | 3.8-5.9 | 2.1-3.0 |
| P Value | 0.97 | 0.20 | 0.07 | 0.31 | 0.06 | **0.01** | 0.09 | 0.12 |
| IFIT1 | | | | | | | | |
| Fold Exp | 1.0 | 18.7 | 27.3 | 2.8 | 21.7 | 13.7 | 2.3 | 1.9 |
| Range | 1.0-1.0 | 8.9-39.3 | 21.7-34.4 | 2.3-3.3 | 15.6-30.3 | 9.8-19.1 | 2.2-2.5 | 1.6-2.2 |
| P Value | 0.99 | 0.16 | **0.04** | 0.11 | 0.07 | 0.08 | **0.04** | 0.15 |
| OASL1 | | | | | | | | |
| Fold Exp | 1.0 | 5.3 | 9.0 | 1.5 | 10.0 | 4.6 | 1.9 | 1.4 |
| Range | 0.9-1.1 | 3.2-8.9 | 8.7-9.4 | 1.5-1.6 | 8.2-12.1 | 3.3-6.5 | 1.7-2.0 | 1.2-1.6 |
| P Value | 0.96 | 0.19 | **0.01** | 0.06 | **0.05** | 0.14 | 0.08 | 0.27 |
| OASL2 | | | | | | | | |
| Fold Exp | 1.0 | 2.5 | 3.5 | 3.4 | 8.6 | 8.3 | 2.9 | 1.9 |
| Range | 0.7-1.3 | 1.4-4.5 | 2.9-4.3 | 3.0-3.9 | 8.0-9.2 | 7.0-10.0 | 2.8-2.9 | 1.7-2.1 |
| P Value | 0.90 | 0.36 | 0.10 | 0.06 | **0.02** | **0.05** | **0.00** | 0.11 |
| LY6E | | | | | | | | |
| Fold Exp | 1.0 | 1.2 | 1.1 | 1.9 | 2.3 | 2.1 | 1.2 | 1.3 |
| Range | 0.9-1.1 | 1.1-1.2 | 1.0-1.2 | 1.6-2.2 | 2.3-2.3 | 2.0-2.3 | 1.1-1.3 | 1.2-1.3 |
| P Value | 0.96 | 0.10 | 0.37 | 0.15 | **0.01** | 0.06 | 0.22 | 0.10 |
| MX2 | | | | | | | | |
| Fold Exp | 1.0 | 31.8 | 45.3 | 1.9 | 10.9 | 7.3 | 2.3 | 3.5 |
| Range | 1.0-1.0 | 22.2-45.6 | 31.7-64.8 | 0.9-4.0 | 9.6-12.5 | 6.6-8.0 | 2.0-2.8 | 3.4-3.6 |
| P Value | 0.99 | 0.07 | 0.06 | 0.52 | **0.04** | **0.03** | 0.12 | **0.02** |
| OAS2 | | | | | | | | |
| Fold Exp | 1.0 | 3.4 | 3.6 | 5.8 | 10.8 | 6.0 | 1.5 | 1.7 |
| Range | 1.0-1.0 | 2.8-4.0 | 2.4-5.5 | 5.5-6.0 | 9.4-12.5 | 4.5-8.2 | 1.2-1.9 | 1.2-2.4 |
| P Value | 1.00 | 0.10 | 0.20 | **0.01** | **0.04** | 0.11 | 0.37 | 0.35 |
| IFI44 | | | | | | | | |
| Fold Exp | 1.0 | 1.8 | 2.8 | 0.8 | 3.3 | 5.6 | 2.3 | 1.6 |
| Range | 0.9-1.1 | 1.2-2.9 | 2.5-3.0 | 0.7-1.0 | 2.9-3.8 | 5.0-6.4 | 2.2-2.5 | 1.5-1.6 |
| P Value | 0.96 | 0.41 | **0.05** | 0.46 | 0.08 | **0.05** | **0.05** | **0.04** |

Genes inducible by Type I IFNs were significantly up-regulated at 3-6 hours and again at 2-3 days after inoculation.
